# PathCase-SB architecture and database design

**DOI:** 10.1186/1752-0509-5-188

**Published:** 2011-11-09

**Authors:** Ali Cakmak, Xinjian Qi, Sarp A Coskun, Mitali Das, En Cheng, A Ercument Cicek, Nicola Lai, Gultekin Ozsoyoglu, Z Meral Ozsoyoglu

**Affiliations:** 1 Electrical Engineering and Computer Science Department, Case Western Reserve University, 10900 Euclid Ave., Cleveland, 44106, OH USA; 2 Department of Biomedical Engineering, Case Western Reserve University, 10900 Euclid Ave., Cleveland, 44106, OH USA; 3 Department of Pediatrics, Case Western Reserve University, 10900 Euclid Ave., Cleveland, 44106, OH USA

## Abstract

**Background:**

Integration of metabolic pathways resources and regulatory metabolic network models, and deploying new tools on the integrated platform can help perform more effective and more efficient systems biology research on understanding the regulation in metabolic networks. Therefore, the tasks of (a) integrating under a single database environment regulatory metabolic networks and existing models, and (b) building tools to help with modeling and analysis are desirable and intellectually challenging computational tasks.

**Description:**

PathCase Systems Biology (PathCase-SB) is built and released. The PathCase-SB database provides data and API for multiple user interfaces and software tools. The current PathCase-SB system provides a database-enabled framework and web-based computational tools towards facilitating the development of kinetic models for biological systems. PathCase-SB aims to integrate data of selected biological data sources on the web (currently, BioModels database and KEGG), and to provide more powerful and/or new capabilities via the new web-based integrative framework. This paper describes architecture and database design issues encountered in PathCase-SB's design and implementation, and presents the current design of PathCase-SB's architecture and database.

**Conclusions:**

PathCase-SB architecture and database provide a highly extensible and scalable environment with easy and fast (real-time) access to the data in the database. PathCase-SB itself is already being used by researchers across the world.

## Background

There are many computer science applications that integrate the data of different data sources, and build new tools that are otherwise difficult or impossible to build. PathCase Systems Biology (PathCase-SB) [1] brings together, under a single database environment, metabolic pathways data and systems biology models, and provides new or expanded browsing, querying, visualization, and simulation capabilities in order to help with systems biology modeling and analysis, all brought about due to the integrated environment. Note that PathCase-SB, which builds on PathCase [2], is not a model- or pathways-data source, and, it does not curate systems biology models. In this paper, we describe architecture and database design issues encountered in PathCase-SB's design and implementation, and present the current design of the PathCase-SB architecture and database. The user interfaces of PathCase-SB are described in detail in another study (Coskun et al: PathCase-SB: Integrating Data Sources and Providing Tools for Systems Biology Research, submitted).

PathCase-SB has two goals:

1. Integrate into a single database selected data of systems biology data sources *and *biochemical network data sources, and

2. Build a database-enabled and SBML- [3] or CellML-centered [4,5] visualization, querying, simulation, and comparison and analysis environment for systems biology models and the biochemical networks that they model--with the goal of *providing added-value *as compared to the original data sources.

The current version of PathCase-SB, released on August 2010, integrates models from BioModels Database [6-8] and KEGG pathways [9], and has four user interfaces, namely, Browser, Querying, and Visualization interfaces for exploratory search, querying, and visualization, and the Simulation Interface to comparatively simulate models; more details about the user interfaces are described elsewhere (Coskun et al: PathCase-SB: Integrating Data Sources and Providing Tools for Systems Biology Research, submitted). Additional user interfaces are currently being designed and implemented.

This paper is about the architecture and database design of PathCase-SB. More specifically, we describe

• Data source integration issues encountered during PathCase-SB design, and the approach taken to resolve them,

• BioModels Database SBML parser and KEGG parser developed for the original data sources, and problems encountered,

• Current PathCase-SB architecture and database design, and the resulting database schema, and

• Current database content.

BioModels Database and CellML repositories are two leading open-access primary data sources that biologists and modelers use to store, search, exchange, and retrieve published quantitative systems biology models. Models from BioModels Database are available as SBML documents which are enriched with additional annotations in RDF (Resource Description Framework [10]) format. CellML models are available as CellML documents. Both SBML and CellML are broadly accepted markup languages for representing systems biology models. PathCase-SB currently imports from BioModels Database (and soon, CellML) and KEGG; however, we are also exploring the possibility of incorporating Reactome [11] pathways into PathCase-SB database.

PathCase-SB architecture and database are designed with four *performance goals *in mind.

• *Extensibility*. The database is currently populated with three parsers, namely, BioModels Database SBML parser, CellML parser, and KEGG FTP data parser. Since biological data sources continually extend or change (i.e., improve or completely redesign) their data formats, these three PathCase-SB parsers also continually go through major or minor revisions. Accordingly, (a) the corresponding PathCase database data model is designed to accommodate such (sometimes simple and other times, significant) changes with relative ease, and (b) all visualization and graph manipulations are encapsulated within a client-side applet, and delegated to client-side-only computation. In summary, the PathCase-SB architecture and database are designed with extensibility in mind, i.e., the design takes into consideration possible future growth and changes.

• *Easy data access*. User interfaces of computational applications for life sciences are usually very rich and detailed with many types of data. In order to allow such interfaces provide fast and easy data access for many different user interface functionalities, the database designs of applications should be in synch with their user interface designs, which, we hope, is the case for PathCase-SB. And, in addition, PathCase-SB interfaces are multi-faceted, involving sophisticated browsing, querying, visualization, and simulation components. PathCase-SB database is designed with easy access in mind for all the functionalities of PathCase-SB user interfaces.

• *Fast (real-time) response time for all interface functionalities*. PathCase-SB tools are available online to users on the web. Consequently, the architecture and database is designed for providing real-time response capabilities for each functionality in PathCase-SB user interfaces. Visualization interface is designed to minimize client-server trips by shipping the original visualization specifications from the server to the client (because today's personal computers and workstations have significant computing power and memory) only once, and then performing all visualization commands only at the client side, with no communications with the server. The design takes into account the requirement that all PathCase-SB queries must be processable in real-time, i.e., in a matter of seconds. That said, in some cases, this requirement also necessitates main-memory-based maintenance and manipulation of subsets of PathCase-SB database tables. We are currently looking into rewriting some of the user interface code (namely, browser interface) which cashes parts of database tables and retains them in main memory, in order to speed up observed response-time delays of some interface functions.

• *Scalability*. PathCase-SB can be accessed by a large number of users at the same time, and the PathCase architecture and database must scale effortlessly to concurrent accesses by hundreds of users. Fully completed stress tests on PathCase-SB will be reported elsewhere; however, by load balancing on two fast servers and by offloading visualization to the client side, PathCase-SB scales to concurrent access of many (low hundreds) users.

### Construction and content

In this section, we briefly summarize the PathCase-SB system architecture and database content.

### System Architecture

**Figure 1:**
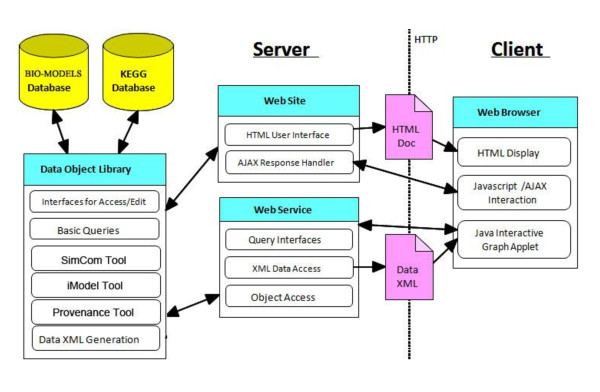
**PathCase-SB Architecture**.

PathCase is a series of applications developed in Microsoft.NET that serve a wide variety of purposes. This paper discusses the database of one of the applications, namely, PathCase-SB. Current PathCase-SB architecture is shown in Figure 1 (CellML part is not yet available to the public, and is omitted). As is generally the case in web applications, PathCase-SB is a three-tiered system comprised of a database, server applications, and multiple client-side interfaces that communicate with the server via HTTP.

At the server-side, PathCase-SB data is managed by a relational database management system, namely, Microsoft SQL Server 2008. In addition, an object-oriented data-access interface between the relational database and the application layer is provided in the form of a large set of wrapper class functions, in order to provide easy, extensible, and fast data access as well as to prevent major changes in the application when a schema change occurs during the evolution of PathCase-SB. When designing the object model behind the system, a persistent object model is used which hides the details of the actual database implementation and database access. As an example, users can easily create an object that can be stored into the database or search for a specific object by using the PathCase-SB API (application programmer's interface) without writing any SQL queries.

For any browser functionality that requests a new visualization, the server first prepares an XML document providing all visualization details, and sends it to the Graph Viewer (applet) at the client-side. The Graph Viewer visualizes the data, and, handles all subsequent interactive visualization-related user commands of the current visualization directly and without any client-server communication. This speeds up the command execution, and scales the visualization. On the flip-side, if the client machine is old and slow, or is short in main memory, PathCase-SB visualizations also slow down, but, from our own testing, a typical average office PC/laptop can easily run PathCase-SB visualization tools with no noticeable performance problems.

Client-side PathCase-SB functionality includes basic HTML that renders the main site interface to the user, and JavaScript with AJAX that makes the site highly responsive to the user.

### Database Content

The amount of core data in PathCase-SB database, which is parsed from BioModels Database and KEGG, has been updated periodically since its initial version 1 (2008) to the current release (Version 2, December 2010). From Version 1 to Version 2, PathCase-SB database has grown 200 MB in size, and nine new tables are added to the database schema. As a result, Version 2 of the PathCase-SB database contains 20% more models, 37% more reactions, 10% more processes, 4% more basic molecules, and ~4% more proteins when compared to PathCase-SB database in 2008. The detailed statistics are shown in Table 1.

**Table 1:** Comparison of PathCase-SB database content between 2008 and 2010

Item Type	Quantity in 2008	Quantity in V 5.0	Percentage Increase
**Models**	209	252	20.57416268

**Reactions**	3787	5189	37.02138896

**Processes**	6990	7672	9.756795422

**Basic Molecules**	26149	27220	4.09575892

**Proteins**	4994	5191	3.94473368

## Utility and Discussion

Please note that the user interfaces of PathCase-SB system are extensive, covered in a separate paper (Coskun et al: PathCase-SB: Integrating Data Sources and Providing Tools for Systems Biology Research, submitted), and will not be discussed here. Next, in the section on "data model design", we discuss data model design issues. The section on "scalability and efficiency" discusses two optimization schemes for browsing and built-in query processing, namely, the use of labeling schemes and caching small dimension tables for main-memory joins. The section on "BioModels Database SBML parser" presents the details of parsing SBML documents from the BioModels Database site. In the section on "capturing BioModels information in PathCase-SB database", we discuss how BioModels Database data is converted into PathCase-SB database schema (CellML part of PathCase-SB database and the associated parser are functional, but not discussed to save space). The section on "mappings" presents how BioModels SBML documents "link" models to external data sources; and, based on this knowledge, in the section on "PathCase-SB Database Schema", we discuss the actual mapping- and annotation-related tables of PathCase-SB.

**Figure 2:**
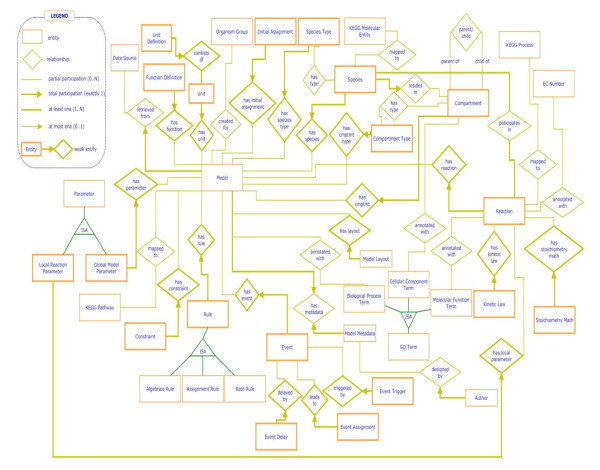
**Systems biology-related component of PathCase-SB database entity-relationship diagram**.

**Figure 3:**
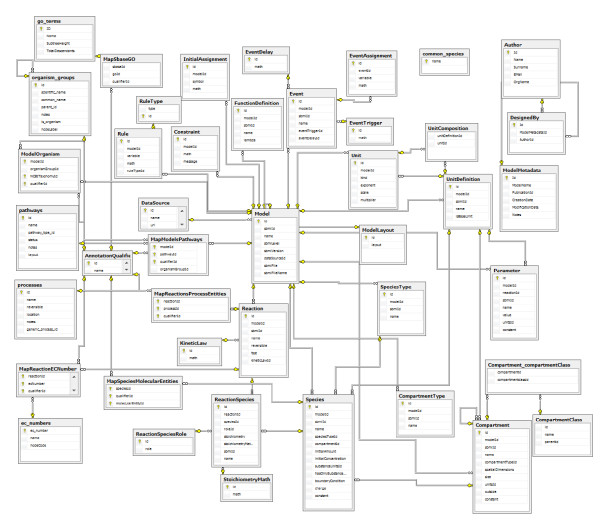
**System Biology-related part of PathCase-SB Database Schema**.

**Figure 4:**
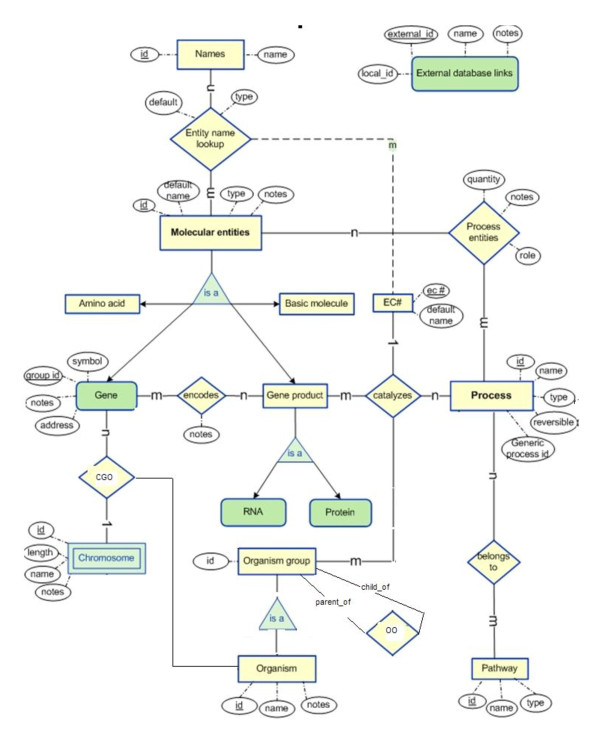
**Classes and the relationships between biological entities via the entity-relationship model**.

Appendix 1, Figure 2 presents the ER-diagram of the BioModels-related components of PathCase-SB. Appendix 2, Figure 3 contains the corresponding PathCase-SB database schema. In Appendix 3, Figure 4, we present the E-R diagram for KEGG-related parts of PathCase-SB database schema. Finally, Appendix 4 summarizes the BioModels qualifiers.

### Data Model Design

One design criterion used is for PathCase-SB never to involve any data curation. With this in mind, PathCase-SB database design has encountered two data integration choices, discussed next.

#### Tightly or loosely coupled integration of data from sources of **different **types

One design choice is whether or not to integrate common information between systems biology (e.g., BioModels) data sources and biochemical reaction network data sources (e.g., KEGG). In an earlier database design, such information was fully integrated, and soon many data integration, data repopulation, and curation problems surfaced. The current approach for capturing data that originate from different systems biology and biochemical network databases is to have *separate tables *for common entities of different systems biology and/or biochemical network data sources, e.g., BioModels Database and KEGG data sources. As an example, PathCase-SB database maintains a *species *table occurring in BioModels Database models and *molecular_entities *table for KEGG molecular entities. Such an approach allows the system to cleanly separate data from different data sources, and, to add new data sources seamlessly, without any future data cleansing and data integration problems. The current approach however requires an additional "mapping" effort: PathCase-SB maintains mappings between all pairs of data sources on their corresponding entities. For BioModels and KEGG, there are three major mappings: <*species, molecular entities*>, <*reactions, process-entities*>, *<models, pathways>*.

#### Tightly or loosely coupled integration of data from sources of **similar **types

A second design choice is whether or not to integrate data in *different *systems biology (or metabolic network) data sources into a single set of tables. As it turns out, the data models of SBML-oriented data sources (such as BioModels) and CellML data source are significantly different, turning any tightly-coupled data integration into a significant data curation issue. Thus, at this point in time, PathCase-SB keeps separate tables for different systems biology data sources as well. As an example, currently, PathCase-SB generates and maintains distinct sets of tables for BioModels Database and CellML data sources.

Briefly, the advantages of keeping separate tables for different systems biology data sources and pathways data sources are as follows.

• *No data curation*. Integrating data from different systems biology data sources almost always translates into further data curation, which may be prone to errors, and needs extensive domain expertise. The current PathCase-SB database design approach avoids data curation as a design principle.

• *Simple and easy to maintain application*. The clean separation of data originating from different data sources, and creating selective "mappings" is a simpler and easier-to-maintain approach, and is used by PathCase-SB. The interfaces of PathCase-SB are designed in such a way that data in different data sources are naturally presented separately, while model data is linked to biochemical network data (e.g., KEGG) and relevant annotations (e.g., GO and SBO annotations) at the presentation level on an as-needed basis.

• *Easy data replenishment*. With a data separation-and-mapping approach, replenishing data from a data source becomes a relatively straightforward work, in comparison to the task of curating and integrating data of different data sources with each data reload.

• *Easier adaptation to data source format/version changes*. The exception to "easy data replenishment" occurs when, for various reasons, the data source itself makes significant format/version changes that translate into significant data source parser changes on the PathCase-SB side, which unfortunately occurs sometimes. We have already encountered such data source format/version changes when both BioModels Database and KEGG data formats have changed recently--BioModels Database changes involving relatively easy-to-handle SBML version improvements, and KEGG changes involving significant format changes, with an overall effect of significant changes in our parser applications. Nevertheless, if the data of PathCase-SB were to be tightly coupled, we believe that PathCase-SB database repopulation would have been even more difficult.

At the present time, PathCase-SB database has data from the BioModels Database (as the systems biology data source), and from the KEGG data source (as a metabolic pathways data source).

Currently, the PathCase-SB database contains four classes of tables capturing the following information:

**a. Biochemical reaction network-related tables**: These tables capture information in a biochemical reaction network as defined by a well-defined web-based data source on biochemical networks. As the biochemical network and its database, we use the KEGG metabolic pathways and the PathCase database (see [12]). The PathCase metabolic pathways database is published [12], and we use it as-is in the PathCase-SB. For more details, please see the online PathCase site [13] and its data model [12].

**b. Systems biology data source-related tables: **These tables capture information in a systems biology models data source. This paper presents only the tables for the BioModels Database. The information captured in a model includes quantitative kinetic information, dynamic behavior, the involved species, reactions, etc.

**c. Tables mapping data from different data sources: **These are a set of *mapping tables *that map biochemical network information (e.g., KEGG pathways) to the systems biology models (e.g., the BioModels Database), and

**d. Tables annotating systems biology data with other ontologies or taxonomies: **These tables capture annotations of systems biology models (e.g., as found in the BioModels Database SBML documents).

### Effects of Scalability and Efficiency Requirements on the Data Model Design

Towards the goals of improving time efficiency and scalability, the PathCase-SB system has utilized two optimization schemes for browsing and built-in query processing, namely, the use of labeling schemes for efficiently evaluating descendant/ancestor queries within hierarchical structures such as Gene Ontology (GO) [14], and the use of caching small dimension tables to avoid multiple disk-based joins during query processing.

### Labeling Schemes for Descendant/Ancestor Queries Involving Gene Ontology

The Gene Ontology is the result of an effort aimed at providing a controlled vocabulary for describing roles of genes and gene products in any organism. Three ontologies, *biological process*, *cellular component*, and *molecular *function, are defined in GO. They are represented as directed acyclic graphs (DAGs) in which the terms form nodes and the relations between terms form edges.

The use of labeling schemes when traversing the Gene Ontology helps improve the performance of the PathCase-SB browser when browsing for "BioModels Database models by GO terms". More specifically, the PathCase-SB browser functionality "Browse BioModels Database models by GO terms" takes a GO term *t*, obtains all descendant terms of the term *t *within the GO, and lists all models that refer to terms in all descendants of *t *as well as the term *t*. This functionality can be implemented in an iterative manner, which becomes time-inefficient (and, thus, does not scale well).

To overcome the limitations of traditional iterative methods for descendant/ancestor query evaluation, we have chosen to use a labeling scheme for modeling DAG-shaped hierarchies (such as GO) in order to efficiently process descendant/ancestor queries. In PathCase-SB, we use NodeCodes labeling [15,16] for querying taxonomy annotated entities, such as models, organisms, and biological compartments. NodeCodes is a graph encoding scheme originally proposed for encoding single source directed graphs [15]. First, the roots (nodes with in-degree 0) are labeled (we may consider adding a "virtual" source node *s *and making all roots the children of *s*). For each node *u *in the graph, the set of NodeCodes of *u*, denoted NC(*u*), are assigned using a breadth-first-search traversal starting from the source node as follows: If *u *is the virtual source node, then NC(*u*) contains only one element, the empty string. Let *u *be a node with a set of NodeCodes, NC(*u*), and *v_0_*, *v_1_*,...*v_k _*be *u*'s children in sibling order, then for each *x *in NC(*u*), a code *xi *is added to NC(*v_i_*), where 0 ≤ *I *≤ *k*. While we have chosen to use the NodeCodes labeling in PathCase-SB, there are also interval-based labeling and prefix-based labeling schemes, which are efficient for evaluating descendant/ancestor queries in GO as well. Next we briefly evaluate the time efficiency of using the NodeCodes labeling approach in PathCase-SB.

As of Aug. 2010, Gene Ontology has 10,268 terms having one or more descendants. In our first experiment on time efficiency, we have run descendant queries for these 10,268 terms, and compared two methods: *Iterative *and *NodeCodes*. The *Iterative *method refers to the method where the descendants of a given term are retrieved by iteratively traversing the GO graph. The *Iterative *method is our baseline approach which is commonly utilized by existing tools [17,18]. The results are shown in Table 2 in terms of the average query processing time for 10268 terms from each method. From Table 2, the *NodeCodes *labeling method performs 2.50 times faster than the *Iterative *method.

**Table 2:** Average query processing time for 10,268 terms having at least one descendant

Methods	Average query processing time (ms)
**Iterative**	244.39

**NodeCodes**	97.68

In our next experiment, we have run descendant queries on five different classes: each class corresponds to a different choice of input node type, and, therefore, of different query selectivity. Here, query *selectivity *is defined as the proportion of terms that it returns. In Gene Ontology, the term (GO: 0008150) *biological_process *has the maximum number of descendants. This term has 14,889 descendants, which leads to the maximum selectivity (58.7%) for the descendant query. In this experiment, we have chosen to use selectivity values ranging from 58.7% to 0.01%. We have run these five query types 50 times each, and the average query processing time is shown in Table 3.

**Table 3:** Execution time of descendant queries for five different query classes of differing selectivities (% selectivity is defined as the proportion of terms that it returns)

Methods	Class 1	Class 2	Class 3	Class 4	Class 5
**Selectivity**	58.7%	22.1%	4.96%	1.27%	0.01%

**Iterative (ms)**	125031.25	47687.50	10359.38	2656.25	31.25

**NodeCodes (ms)**	656.25	484.38	609.38	437.50	531.25

**Iterative/NodeCodes**	190.52	90.45	16.99	6.07	0.05

**Figure 5:**
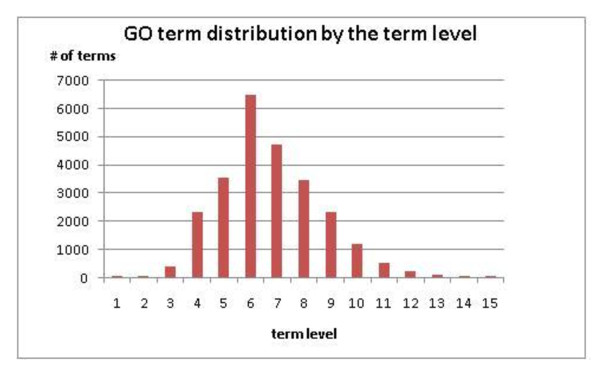
**Go term distribution by the term level**.

From Table 3, *NodeCodes *requires close to constant time for processing each class. For Class 1 and Class 2, *NodeCodes *Labeling performs 190.52 times and 90.45 times faster than the *Iterative *method, respectively. Figure 5 shows the distribution of terms by the term level in the GO hierarchy.

### Caching Small Dimension Tables for Main-Memory Joins

PathCase-SB utilizes a caching scheme for optimizing browsing and built-in query processing. More specifically, for fact-dimension tables, PathCase-SB caches all small dimension tables (e.g., reaction participant roles) in the database subschemas to avoid multiple disk-based joins with the fact table (e.g., reaction participants) in that subschema. In total, there are twelve such cached tables, which include *Attribute*, *EntityName*, *GraphNode*, *MolecularEntityType*, *NameId*, *NameType*, *PathwaysType*, *ProcessEntityRole*, *ReactionSpeciesRole*, *RnaType*, *RuleType*, and *UnitDefiniton*Tables. As an example, consider the *ReactionSpeciesRole *table. The cached table is utilized by four built-in queries:

1) *Find models containing different expressions of metabolic flux associated to the same reaction*;

2) *Find models that contain metabolites of a given pathway*;

3) *Find models that contain reactions of a given pathway*; and

4) *Find kinetic models corresponding to a given reaction in a given pathway*.

In the third query "Find models that contain reactions of a given pathway", the tabular results include *Species Name *(*Reaction Role*) column which is obtained from a join between *ReactionSpecies *and *ReactionSpeciesRole *tables on the column *roleId *from *ReactionSpecies *table. Since joins are expensive, and such a caching approach improves the efficiency of query processing, our caching approach helps with PathCase-SB real-time query processing and scalability requirements.

### BioModels Database SBML Parser

**Figure 6:**
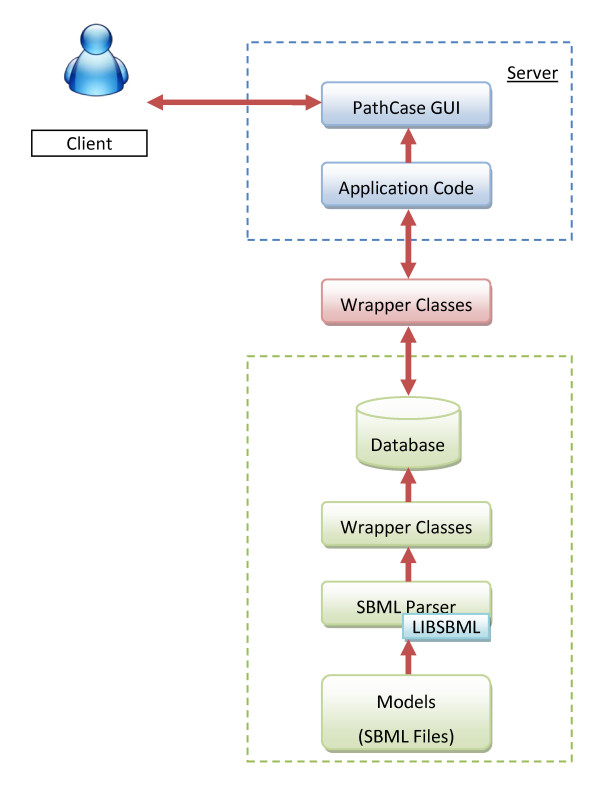
**Flowchart for SBML parser and its application**.

The chart in Figure 6 illustrates the data flow of the BioModels Database SBML parser application, and the PathCase-SB tools that use the database. The parser parses each element of the SBML document by employing the libSBML library API [19,20], which in turn contains function calls for reading, writing, manipulating, and validating content expressed in SBML format.

After parsing the relevant SBML elements or annotation, the parser populates the database not by direct inline SQL calls, but by employing a large number (about 200) of database wrapper class functions. One problem with having inline SQL code is that, since there is no abstraction from the data source, one is bound to a particular schema because table and column names are specified directly in the code, and, this may have negative implications: (i) when the data source is changed, the whole parser application code needs to be scanned, and the corresponding inline SQL calls need to be changed one by one, instead of revising just the wrapper class library; and (ii) error handling becomes difficult and decentralized.

PathCase-SB system uses object-oriented wrapper classes to access the data. Wrapper classes share common interface and data access code, as well as common utilities such as logging (which is implemented, but not currently made available) and error handling. The new class library is called *PathwaysSBLib*. There are two classes that all database access classes use, *DBWrapper *and *DBRow*. *DBWrapper *implements a singleton pattern via the use of an instance, and creates one single database connection at a time, which is shared by multiple database calls. *DBRow *is used to abstract the idea of a database tuple (i.e., row) into a class in an object-oriented manner. These two classes centralize all database access through a series of public functions. All pertinent information such as *connection string *(which is stored in configuration files) and exception handling are processed inside these classes. This approach of "single point of database contact" allows us to centralize all data access information and customization, making future updates much easier.

Each table in the PathCase-SB schema has a "*Server*" wrapper class associated with it. Each one of these classes exposes public properties that correspond to columns in that table. This approach allows us to change the database schema without breaking an existing class that might reference the class at hand. It also allows for *type-safe access to data *without any knowledge of column names or database schema. Type-safe access means, for example, that the code specifies in property declaration that ID is an *integer)*, compared to inline SQL usage where one would cast a column to a data type without any guarantees that it will work.

### Capturing BioModels-Based Model Information in PathCase-SB Database

In Section "BioModels Database Entities and Relationships", we present entities and relationships of data in the BioModels Database data source. Section "PathCase-SB Schema: BioModels Database-Related Information" lists database table schemata, and presents additional details for each table. Since BioModels Database uses SBML documents to capture its data, we view a model from a BioModels Database as an SBML document. A BioModel Database model also captures additional information, mostly in the form of annotations.

### BioModels Database Entities and Relationships

BioModel Database SBML documents capture all entities as defined by the Systems Biology Markup Language (SBML) Level 2, Version 3 specification. SBML is a model representation format for systems biology, and allows models of arbitrary complexity to be represented. More specifically, SBML allows the specification of the following entities: *Model, Species, SpeciesType, Reaction, Compartment, CompartmentType, Event, Rule, RuleType, Constraint, FunctionDefinition, UnitDefinition, Parameter, InitialAssignment*; for more details on these entities, please see the SBML Level 2, Version 3 specification [3].

In addition to SBML-based entities, PathCase-SB has other entities and relationships, namely, *DataSource, Sbase, ModelLayout *(PathCase-SB provides a visualization of the biochemical network whose kinetics is captured by the model. The PathCase-SB *ModelLayout *entity instance represents the layout of the visualization. Note that most PathCase-SB network visualizations are created automatically by graph layout algorithms. However, for more complicated model networks, sometimes, visualizations are manually beautified and "frozen", and the *ModelLayout *entity retains the details of the frozen layout)*, KineticLaw, ReactionSpeciesRole, ReactionSpecies, StoichiometryMath, EventTrigger, EventDelay, EventAssignment, EventAssignmentArgument*, and *KineticLawParameter*. To save space, below, we describe only a few of these PathCase-SB-specific entities and relationships. Please see Appendix 1, Figure 2, for the ER diagram.

**DataSource entity **retains all information specific to a systems biology data source.

**Sbase **entity serves as a base object for the actual entities. Each entity (except *DataSource*) in the database extends *Sbase *entity. Sbase contains a sboTerm attribute for SBO (*Systems Biology Ontology*) annotations. Most SBML elements have SBO annotations which define the semantics of the corresponding term. For instance, for a modifier in a reaction, its SBO term (if available) may specify its specific modifier role (e.g., inhibitor, catalyzer, and regulator) in that reaction. Storing SBO annotations helps in terms of establishing connections both between different models and between metabolic pathways and models.

**Model **entity contains basic information for each systems biology model.

**StoichiometryMath **entity specifies the full stoichiometry of a given reaction. SBML specification allows the definition of stoichiometry through either double values, or MathML expressions (for more complicated stoichiometry representation). This entity includes two stoichiometry-related fields: (i) *stoichiometry (float)*, and (ii) *stoichiometryMath (mathml*) where the expression in parentheses shows the type of the stored information.

### PathCase-SB Database Schema: BioModels-Related Information

In this section, we specify the database tables of the BioModels-related part of PathCase-SB database, omitting the CellML- and KEGG-related tables in PathCase-SB. The complete list of BioModels-related tables in the database include *DataSource, Sbase, Model, ModelLayout, FunctionDefinition, UnitDefinition, Unit, UnitComposition, CompartmentType, SpeciesType, Compartment, CompartmentClass, CompartmentClassDictionary, Species, Common_Species, Parameter, InitialAssignment, RuleType, Rule, Constraint, Reaction, KineticLaw, ReactionSpeciesRole, ReactionSpecies, StoichiometryMath, Event, EventTrigger, EventDelay, EventAssignment, Author, ModelMetadata, DesignedBy*. Please see Appendix 2, Figure 3, for the schema diagram.

For ease of understanding, in naming tables, we use the same naming conventions as defined by the SBML Level 2, Version 3, Release 2 specification [3] as much as possible. For explanations of table/attribute names, please see the SBML specifications [3]. Once again, below we list only some of the tables, where each table schema is followed by a brief discussion, if needed, specifying attributes of the corresponding relations, domains, etc. For database terminology such as primary, candidate, foreign key, etc., we refer the reader to standard database textbooks (e.g., [21]). Underlined attributes in each relation schema form the primary key for the relation.

**DataSource **(id, name, url) table captures data source information. *name *is a key (not primary key, but a "candidate" key) in this table.

**Sbase **(id, metaId, sboTerm, notes, annotation). Unless noted otherwise, each entity (except *DataSource*) in this schema extends *Sbase *entity. Therefore, in each table (including *Model *table), *id *field is a foreign key referring to the *id *field in *Sbase *table. Whenever a table contains a field with name "*id*", it is a primary key in that table. Unless noted otherwise, all foreign keys adopt "ON DELETE CASCADE" property.

**Model **(*id, sbmlId, name, sbmlLevel, sbmlVersion, dataSourceId, sbmlFile*) table captures basic information for each systems biology model. *dataSourceId *is a foreign key referring to the *id *field in *DataSource*table.

**ModelLayout **(*id, layout*) table. One lesson we learned in PathCase [2] is that automated biochemical network layouts are not satisfactory for life scientists as they become familiar with the same specific pathway layouts (e.g., the TCA cycle layout with Acetyl CoA at the top of the layout) and would like to see the same pathway layouts within a larger network. Therefore, in PathCase-SB also, we manually curate pathway/biochemical network layouts, and always use the same curated layout for visualizations. This table maintains manually curated biochemical network visualization layout information for the system biology model identified by *id*, where *id *is a foreign key referring to the id field in *Model *table.

**Compartment **(*id, modelId, sbmlId, name, compartmentTypeId, spatialDimensions, size, unitsId, compartmentClassId, outside, constant*).

**CompartmentClass **(*id, name, parentId*) table contains a biologically well-defined compartment classification class hierarchy. *parentId *is a foreign key referring to the id field in *CompartmentClass *table. Parent of root compartments is *null*.

**CompartmentClassDictionary **(*compartmentName, compartmentClassId*) table holds information needed to match the correct compartment class with a compartment. This table is mainly used by SBML parser during the creation of each compartment. Compartment names in this table are unique.

**Species **(*id, modelId, sbmlId, name, speciesTypeId, compartmentId, initialAmount, initialConcentration, substanceUnitsId, hasOnlySubstanceUnits, boundaryCondition, charge, constant*) table captures species information and the relationships it is involved in. Attribute *speciesTypeId *is a foreign key referring to the id field in *SpeciesType *table; *substanceUnitsId *is a foreign key referring to the id field in *UnitDefinition *table; and *compartmentId *is a foreign key referring to the id field in *Compartment *table.

**Reaction **(*id, modelId, sbmlId, name, reversible, fast, kineticLawId*) table captures reaction information in models.

**ReactionSpecies **(*id, reactionId, speciesId, roleId, stoichiometry, stoichiometryMathId, sbmlId, name*) table captures species information in reactions.

**Author**(*id, name, surname, email, orgName*) table contains general information about authors.

**DesignedBy**(*id, modelKey, authorKey*) table captures information about the creator(s) of each model. It represents many-to-many relationship between Model and Author tables which are linked through *modelKey *and *authorKey *foreign keys, respectively.

**ModelMetadata **(*id, modelName, publicationId, creationDate, modificationDate, notes*) table contains version (date) information about the model. *publicationId *is a foreign key which is used to link the *Model *to BioModels[6] original web site for detailed version history of the model.

### More on SBML-to-Database Conversion

Below we briefly discuss a number of additional issues that have arisen when pushing data in SBML documents into database tables.

#### Loss of id information in SBML Models

Each table in a PathCase-SB database needs to have an id field of type *uniqueidentifier*. On the other hand, each SBML model specification contains an id field of type *alphanumeric *string for the corresponding entities. Ids in an SBML model are used for cross-referencing among different entity definitions, and, most of the time, id is the only meaningful name (e.g., pow3 for a function definition representing x^3^) for the corresponding entity. Although each database table has an id field, such fields store an arbitrarily generated unique identifier which is independent of the corresponding model. This information is usually crucial, especially when models omit the optional name field, and use id for that purpose. Thus, we have added the *sbmlId *field into the corresponding relations, for which SBML specification defines an id field.

#### Accommodating Function-Mathematical Expression Connection

Most of the mathematical expressions (represented in MathML) such as kinetic laws, rules, etc., in a model are allowed to contain references to pre-defined mathematical functions in the model. To accommodate such a relationship in the database model, one alternative is to consider it as an argument like species, parameter, and compartment names in math expressions. And, adding an attribute function_id into the argument entity accommodates references to function names in different MathML expressions throughout the model.

#### Revisiting Unit Storage

In SBML, there are built-in "base" units, and user-specified units that are defined based on built-in units. User-specified units cannot be built upon other user-specified units. Furthermore, since user-specified units are defined in the context of a specific model to provide connection between *units *and *models*, model ids can be included as a field in *units *table. For built-in base units, the model id is set as *null*. And, base units are pre-specified before any database population.

### User Model Upload Database

To implement the functionality of users uploading their own models for visualization and simulation, PathCase-SB maintains a separate database, namely, *Pathcase_SB_UserUploads *in order to temporarily save user-uploaded data. This database has a schema similar to the Pathcase-SB schema. The user-uploaded data is presently kept in a separate database than the PathCase-SB database due to (i) privacy and intellectual property rights concerns, and (ii) possible manageability issues to maintain, purge, and integrate the uploaded data into the existing permanent data.

### Mappings between BioModels Database Models and External Databases

Part of the BioModels' curation effort involves linking components of a systems biology model to other external and related databases. Such external links are located in the annotation field of each major entity in an SBML model. Annotation fields in SBML are formatted in RDF (the resource description Framework ([10]. Each such external link entry is annotated with a qualifier (e.g., "is", "isHomologTo", "isDescribedBy", etc.). Such annotations describe the nature of mapping, and are important for users to decide if the mapping is a "perfect match" or a "partial match". Please see Appendix 4 for a brief description of each possible qualifier.

### Example: Glycolysis Models

To have an idea about the completeness of the external links to KEGG database in BioModels Database models, we have studied a large number of BioModels Database models. Table 4 specifies those that are listed under the Gene Ontology (GO) cellular process term "Glycolysis". Our conclusion is that BioModels Database models are well annotated in that, if a reaction or a metabolite in a model has a corresponding reaction in KEGG, it has an external link annotation for KEGG. The observations for the availability of external links in some of the studied models are summarized in Table 4.

**Table 4:** External Link Information in Models

*Model Id*	*External Link to KEGG Pathway*	*External Link to NCBI Organism Taxonomy*	*External Link to GO Biological Process*	*Total Number of Reactions*	*Reactions with KEGG External Link*	*Reactions without KEGG External Link*	*Total Number of Metabolites*	*Metabolites with KEGG External Link*	*Metabolites without KEGG External Link*
**Conant 2007**	Yes	yes	yes	20	15	5	27	26	1

**Bakker 2001**	Yes	yes	yes	14	10	4	13	11	2

**Nielsen 98**	Yes	yes	yes	25	9	16	14	14	0

**Fung 2005**	No	yes	yes	12	3	9	8	5	3

**Hynne 2001**	Yes	yes	yes	22	13	9	24	24	0

**Wolf 2000**	Yes	yes	yes	11	6	5	9	9	0

We make the following additional observations.

• The reactions that do not have an external link to KEGG are almost always non-metabolic reactions which are not in the scope of KEGG. Such reactions are usually flow/transport reactions or interactions that involve genes and/or transcription factors.

• Among the six Glycolysis models in Table 1, only one of them (Fung 2005) does not have an external link to a KEGG pathway. We have looked into the abstract of the corresponding publication for this model, and observed that this work mostly studies the effect of gene expression levels on the fluxes of reactions in Glycolysis, and, hence, was not mapped to a KEGG pathway.

• Some reactions in a model may be mapped to two or more distinct KEGG reactions. Such one-to-many mappings possibly correspond to reactions that are lumped together in the systems biology model at hand.

• Some species in a model may be mapped to two or more distinct KEGG compounds. Such one-to-many mappings possibly correspond to different optical conformations of the same metabolite. As an example, glucose exists in two optical conformations as D-Glucose and L-Glucose depending on the species.

### Links

All of the links summarized in this section are illustrated in Appendix 5 with example entries.

• **Linking Models to Pathways**. The annotation field of each *model *element contains links to five distinct external pathway-related databases, namely, KEGG, Reactome, NCBI's organism taxonomy, PubMed, and Gene Ontology (See Appendix 5 with example entries). Note that some models may have only a subset of the five external links.

• **Linking Reactions**. *The annotation field *of each *reaction *element contains links to four external related databases, namely, KEGG, Reactome, EC number, and Gene Ontology. Note that some reactions may have only a subset of the four external links.

• **Linking Species to Metabolites/Compounds**. The annotation field of each *species *element c**o**ntains links to two external *relate*d databases, namely, KEGG and CHEBI [11,22,23] which are listed in Appendix 5 with example entries. Note that some species may have only a subset of the two external links.

• **Linking Compartments to GO**. The annotation field of each *compartment *element con*tains link *to GO Cellular Component Subontology, which may be used to reconcile compartments from different models.

### PathCase-SB Database Schema: Mappings Between BioModels Database and KEGG Pathways

#### Mapping Tables

We have designed and populated three tables that map BioModels Database entities and KEGG entities:

(a) *Species *of systems biology models and *Molecular Entities *of KEGG,

(b) *Reactions *of systems biology models and *Process Entities *of KEGG, and

(c) *Models *of systems biology models and *Pathways *of KEGG.

Below we briefly specify the schema of the three relations.

**MapSpeciesMolecularEntities **(*speciesId, molecularEntityId, qualifierId*). In this table, *molecularEntityId *is a foreign key referring to the id field in *molecular_entities *table; *speciesId *is a foreign key referring to the *id *field in *species *table, and *qualifierId *is a foreign key referring to the *id *field in *AnnotationQualifier *table.

**MapReactionsProcessEntities **(*reactionId, processId, qualifierId*). In this table, *processId *is a foreign key referring to the id field in *processes *table; *reactionId *is a foreign key referring to the id field in *reaction *table; and *qualifierId *is a foreign key referring to the id field in *AnnotationQualifier *table.

**MapModelsPathways **(*modelId, pathwayId, qualifierId, organismGroupId*). *modelId *is a foreign key referring to the id field in *models *table; *pathwayId *is a foreign key referring to the id field in *pathways *table; *qualifierId *is a foreign key referring to the id field in *AnnotationQualifier *table; and *organismGroupId *is a foreign key referring to the id field in *organismGroup *table.

#### Annotation Tables

We maintain three annotation tables for BioModels Database models:

(a) Annotating *models *with *organisms *from the NCBI organism taxonomy,

(b) Annotating *models *with *Gene Ontology *(GO) *terms*,

(c) Annotating *reactions *in *models *with *EC *(Enzyme Commission) *numbers*.

Below we briefly specify the schemata involving the above-listed three mappings.

**ModelOrganism **(*modelId, organismGroupId, NCBITaxonomyId, qualifierId*) table annotates BioModels Database systems biology models with organisms/organism groups (as specified in the NCBI organism taxonomy). This information is not part of SBML document; however, BioModels Database curators have captured it as metadata (annotation). *modelId *is a foreign key referring to the *id *field in model table; *organismGroupId *is a foreign key referring to the id field in *organism_groups *table; *qualifierId *is a foreign key referring to the *id *field in *AnnotationQualifier *table.

**MapSbaseGO **(*sbaseId, goId, qualifierId*) table annotates systems biology models with GO terms.*sbaseId *is a foreign key referring to the id field in *sbase *table. *goId *is a foreign key referring to the id field in *go_terms *table (inherited from the original PathCase schema [12]); and *qualifierId *is a foreign key referring to the id field in *AnnotationQualifier *table.

**MapReactionECNumber **(*reactionId, ecNumber, qualifierId*). *reactionId *is a foreign key referring to the id field in *reaction *table; *ecNumber *is a foreign key referring to the *ec_number *field in *ec_numbers *table (inherited from the original PathCase schema); and *qualifierId *is a foreign key referring to the id field in *AnnotationQualifier *table.

For efficiency purposes involving browser interface functionalities of PathCase-SB, we also maintain three distinct tables, as listed below.

**AnnotationQualifier**(*id, name*). BioModels' MIRIAM specification [24] defines around 10 different qualifiers that describe the nature of mapping between mapped entities from different databases. Such qualifiers are captured in the *AnnotationQualifier *table. *id *is an auto-increment field. This table is pre-filled with the following *name *attribute values: *is, isDescribedBy, encodes, hasPart, hasVersion, isEncodedBy, isHomologTo, isPartOf, isVersionOf, occursIn, unknown*.

**GONodeCodes **(*goId, nodeCode*) is a secondary table which is added to make "Browse Models by GO terms" functionality of the PathCase-SB web interface more efficient. The table stores precomputed NodeCodes (See section on "Labeling Schemes") for GO terms. *goId *is a foreign key referring to the id field in *go_terms *table (inherited from the original PathCase schema [12]).

**ec_numbers **(*ec_number, name, nodeCode*) table is designed to make "Browse Reactions by EC Numbers" functionality of the web interface more efficient through the use of NodeCodes [15]. The table is inherited from the original PathCase database [12], and modified by adding the *nodeCode *attribute.

#### Summary of Mapping Results

Below, we summarize the current results from mapping KEGG pathways to Biomodels Database models based on the annotation information provided in each model. The extracted annotations are stored in the PathCase-SB database. Currently, the PathCase-SB main browsing interface now has four browsing functions: "Models by Name", "Models by KEGG Pathways", "Models by Organisms" and "Models by GO Terms" in order to aid users with faster and intuitive way to browse models.

##### Model-Pathway Mapping

Out of 252 models in the database, (a) 30 models have a reference to a KEGG pathway, and (b) 229 models have a reference to a GO term. Due to the fact that multiple models are mapped to the same pathway (possibly in different organisms), the number of distinct KEGG pathways referred in 30 models is 30. Among 30 referenced KEGG pathways, only 14 of them are metabolic pathways, and the remaining 16 pathways belong to other categories listed in KEGG, such as signaling pathways, gene-regulation pathways, and so on (see http://www.genome.jp/kegg/pathway.html for the complete list of pathway categories in KEGG).

Since our existing pathways database is designed for and stores only metabolic pathways, only 14 distinct pathways from the PathCase KEGG database have at least one mapped Systems Biology model. These 14 pathways are mapped to 30 distinct BioModels Database models (different models mapping to the same pathway). There is no model that refers to a metabolic pathway which is not included in the PathCase KEGG database.

##### Reaction-Process Mapping

The PathCase KEGG database contains 8,182 processes among which 173 processes have at least one mapped reaction in a BioModels Database model. In the database, there are 6,957 reactions stored (extracted from 252 models), and 421 reactions have a mapping to one or more of the above 173 processes.

##### Species-Molecular Entity Mapping

The PathCase KEGG database contains 27,220 molecular entities among which 223 molecular entities have at least one mapped species in a BioModels Database model. In the database, there are 4,963 species stored, and 1110 species have a mapping to one or more of the above 223 molecular entities.

## Conclusions

In this paper, we have described the architecture and database design and modeling issues of PathCase-SB, which integrates data from different systems biology data sources (BioModels Database and CellML) and a metabolic network data source (KEGG).

We believe that integrating data from multiple biology data sources and then providing additional computational tools is a commonly occurring theme in life sciences, and Pathcase-SB is one such example. And, in designing an application architecture and database for such an integrated environment, there are many lessons to be learned, some easily generalizable to different environments (e.g., making use of database wrappers; separating data of different sources and yet integrating their use and presentation, etc.), and some data source instance-specific (e.g., SBML models or KEGG-specific data formats, etc.). There are also tradeoffs involving extensibility, data access, realtime response time, and scalability. This paper, in the context of PathCase-SB, has highlighted a number of lessons and tradeoffs.

## Availability and requirements

PathCase-SB is freely available for use at http://nashua.case.edu/PathwaysSB/Web.

**Operating Systems: **PathCase-SB is accessed from a browser; therefore, it is platform-independent.

**Browsers: **PathCase-SB is extensively tested with browsers Internet Explorer and Mozilla Firefox. It is also tested to a lesser degree with Google Chrome and with Safari browsers.

**Other Requirements: (a) Ajax and JavaScript**. PathCase-SB makes use of Ajax, which is a way of sending data between a web server and client asynchronously with normal page requests. This helps PathCase-SB remain quick and responsive even if there are a substantial amount of data to display at once. For this and other features of the site to work (such as collapsing windows), JavaScript must be enabled in the browser. All modern web browsers such as those mentioned above support JavaScript, but it is possible to have personal security settings in place that prevent JavaScript from running. If certain portions of the site do not appear to be working properly, the user must ensure that JavaScript is enabled and that the browser security settings allow scripting elements to run. **(b) Cookies**. In order to view certain portions of the site correctly PathCase-SB occasionally uses, which are small bits of text stored on a local system's hard drive. Users must ensure that cookies are enabled in the browser in order to get the most out of PathCase-SB. **(c) Java Runtime Environment**. The PathCase-SB Graph Viewer uses the Java software platform. In order to view the applet, version 1.6(also known as version 6) or later of the Java Runtime Environment must be installed on the system from which the viewer is accessed. Users should download and install the latest version of the free plug-in, JRE 1.6 (also known as version 6.0). Some browsers may not allow Java applets to run because of security concerns; if the JRE is installed properly and the Graph Viewer still does not appear, the user should make sure that the browser's security settings allow Java applets (or in the case of Internet Explorer, ActiveX controls). **(d) Monitor Resolution**. While PathCase-SB has been designed to gracefully fit into just about any monitor resolution, it is best viewed at resolutions of 1024 × 768 pixels and up. 800 × 600 and 640 × 480 resolutions will work, too, but the results may not be quite as pleasing to the eye. (e) **Server-side implementation**. Server-side implementation of PathCase-SB is done with ASP.NET Framework using the C#.NET language.

**Database access support and extensibility**. For safety, integrity, and other reasons, the Pathcase-SB application's database is accessed only through its API (Application Programmers Interface), which has about 50 API functions. Third-party application developers can use the existing API functions currently available in the PathCase-SB API to develop new applications. New API functionalities can also be developed if they are needed for third-party developers.

**Any restrictions to use: **PathCase-SB is free to use by academics and non-academics; there are no restrictions.

## Authors' contributions

PathCase-SB architecture and databases design was a collaborative task, led by AC, ZMO, GO, NL, and (late) MC. PathCase-SB implementation was done by AC (both server-side and client-side components), XQ (client-side components, Web API, and version management), SAC (server-side debugging, software testing and deployment), MD (CellML parser), EC (KEGG data parser), and AEC (software testing). Manuscript has been read and approved by all authors and that all authors agree to the submission of the manuscript to BMC Systems Biology journal.

## Appendix 1. PathCase-SB ER Diagram (SB-Related Part)

In Figure 2, we present the systems biology-related component of PathCase-SB database entity-relationship diagram.

## Appendix 2. Systems Biology-Related Component of PathCase-SB Database Schema Diagram

In Figure 3, we present the System Biology-related part of PathCase-SB Database Schema. Note that SBase is a "base" table for all the systems biology related tables. That is, SBase is the parent entity (through IS_A relationship) of all other systems biology entities in the data model. Sbase table is intentionally excluded from Figure 5 to have a more manageable (i.e., less complicated) view of the schema. Figure 5 also excludes majority of the tables that are inherited from the original PathCase database.

## Appendix 3. PathCase Metabolic Pathways Database Schema

In Figure 4, we present the data classes and the relationships between biological entities via the entity-relationship model.

## Appendix 4. BioModels.net qualifiers

Appendix 4 is a summary from the following web page (retrieved on September 2011)

http://www.ebi.ac.uk/compneur-srv/miriam-main/mdb?section=qualifiers

The qualification of an annotation specifies the relation between a model component and its annotation.

### model-qualifiers

**is: **The specified modeling object is the subject of the referenced resource.

**isDescribedBy: **The specified modeling object is described by the referenced resource.

**isDerivedFrom: **The specified modeling object is described by the modeling object in the referenced resource.

### biology-qualifiers

**encodes: **The specified biological entity encodes, directly or transitively, the subject of the referenced resource.

**hasPart: **The specified biological entity includes the subject of the referenced resource, either physically or logically.

**hasProperty: **The subject of the referenced resource is a property of the biological entity represented by the model component.

**hasTaxon: **The biological entity represented by the model element is taxonomically restricted, where the restriction is the subject of the referenced resource.

**hasVersion: **The subject of the referenced resource is a version or an instance of the specified biological entity.

**is: **The biological entity represented by the model component is the subject of the referenced resource. This relation might be used to link a reaction to its exact counterpart in KEGG or Reactome for instance.

**isPropertyOf: **The biological entity represented by the model component is a property of the referenced resource.

**isDescribedBy: **The specified biological entity is described by the referenced resource.

**isEncodedBy: **The specified biological entity is encoded, directly or transitively, by the subject of the referenced resource.

**isHomologTo: **The biological entity represented by the model component is homolog, to the subject of the referenced resource, i.e. they share a common ancestor.

**isPartOf: **The specified biological entity is a physical or logical part of the subject of the referenced resource.

**isVersionOf: **The specified biological entity is a version or an instance of the subject of the referenced resource.

**occursIn: **The specified biological entity represented by the model component takes place in the subject of the reference resource.

## Appendix 5. External Link Examples

### Linking a Model to a Pathway

The annotation field of each *model *element contains links to external pathway-related databases which are listed below with example entries. Note that some models may have only a subset of the external links listed below.

a. KEGG

<bqbiol:is>

<rdf:Bag>

<rdf:li rdf:resource="urn:miriam:kegg.pathway:tbr00010"/>

</rdf:Bag>

</bqbiol:is>

b. Reactome

<bqbiol:isHomologTo>

<rdf:Bag>

<rdf:li rdf:resource="urn:miriam:reactome:REACT_1383"/>

</rdf:Bag>

</bqbiol:isHomologTo>

c. NCBI's Organism Taxonomy

<bqbiol:is>

<rdf:Bag>

<rdf:li rdf:resource="urn:miriam:taxonomy:5691"/>

</rdf:Bag>

</bqbiol:is>

d. PubMed

<bqmodel:isDescribedBy>

<rdf:Bag>

<rdf:li rdf:resource="urn:miriam:pubmed:11415442"/>

</rdf:Bag>

</bqmodel:isDescribedBy>

e. GO

<bqbiol:isVersionOf>

<rdf:Bag>

<rdf:li rdf:resource="urn:miriam:obo.go:GO%3A0006096"/>

</rdf:Bag>

</bqbiol:isVersionOf>

### Linking Reactions

a. KEGG

<bqbiol:is>

<rdf:Bag>

<rdf:li rdf:resource="urn:miriam:kegg.reaction:R00771"/>

</rdf:Bag>

</bqbiol:is>

b. Reactome

<bqbiol:isHomologTo>

<rdf:Bag>

<rdf:li rdf:resource="urn:miriam:reactome:REACT_1164"/>

</rdf:Bag>

</bqbiol:isHomologTo>

c. EC#

<bqbiol:isVersionOf>

<rdf:Bag>

<rdf:li rdf:resource="urn:miriam:ec-code:5.3.1.9"/>

</rdf:Bag>

</bqbiol:isVersionOf>

d. GO

<bqbiol:isVersionOf>

<rdf:Bag>

<rdf:li rdf:resource="urn:miriam:obo.go:GO%3A0046323"/>

</rdf:Bag></bqbiol:isVersionOf>

### Linking Species to Metabolites/Compounds

a. KEGG

<bqbiol:is>

<rdf:Bag>

<rdf:li rdf:resource="urn:miriam:kegg.compound:C00074"/>

</rdf:Bag>

</bqbiol:is>

b. CHEBI

<bqbiol:is>

<rdf:Bag>

<rdf:li rdf:resource="urn:miriam:obo.chebi:CHEBI%3A18021"/>

</rdf:Bag>

</bqbiol:is>

### Linking Compartments

a. GO

<bqbiol:is>

<rdf:Bag>

<rdf:li rdf:resource="urn:miriam:obo.go:GO%3A0005829"/>

</rdf:Bag>

</bqbiol:is>
